# Divergent evolution of *NLR* genes in the genus *Glycine*: impacts of annuals and perennials’ life history strategies

**DOI:** 10.3389/fpls.2024.1383135

**Published:** 2024-07-09

**Authors:** Abu Bakar Sultan, Humera Nawaz, Fozia Saleem, Sehar Nawaz, Muhammad Danial, Romana Iftikhar, Umer Maqsood, Amna Areej, Sidra Shakoor, Nada H. Aljarba, Rizwan Maqbool, Muhammad Rizwan, Saad Serfraz

**Affiliations:** ^1^ Evolutionary Biology Lab, CABB, University of Agriculture Faisalabad, Faisalabad, Pakistan; ^2^ Department of Botany, Division of Science and Technology, University of Education, Lahore, Pakistan; ^3^ Agricultural Biotechnology Division, National Institute for Biotechnology and Genetic Engineering, Faisalabad, Pakistan; ^4^ Department of Biology, College of Science, Princess Nourah Bint Abdulrahman University, Riyadh, Saudi Arabia; ^5^ Department of Agronomy, University of Agriculture, Faisalabad, Pakistan

**Keywords:** *glycine*, perennials, annuals, *NLR* genes, diversification, evolution

## Abstract

Within the family *Fabaceae*, the genus *Glycine* is composed of two subgenera annuals (2n=40) and perennials. This life strategy transition may have differentially affected the evolution of various gene families. Its cultivated species *G. max* has high level of susceptibility to major pathogens including viruses, bacteria and fungi. Understanding nucleotide-binding domain leucine-rich repeat (*NLR*) genes evolution in soybean is of paramount importance due to their central role in plant immunity and their potential in improving disease resistance in soybean cultivars. In this study, we investigated the significance of this annual-perennial transition on the macroevolution of *NLR* genes in the genus *Glycine*. Our results reveal a remarkable distinction between annual species such as *Glycine max* and *Glycine soja*, which exhibit an expanded *NLR*ome compared to perennial species (*G. cyrtoloba, G. stenophita, G. dolichocarpa, G. falcata, G. syndetika, G. latifolia* and *G. tomentella*). Our evolutionary timescale analysis pinpoints recent accelerated gene duplication events for this expansion, which occurred between 0.1 and 0.5 million years ago, driven predominantly by lineage-specific and terminal duplications. In contrast, perennials initially experienced significant contraction during the diploidisation phase following the *Glycine*-specific whole-genome duplication event (~10 million years ago). Despite the reduction in the *NLR*ome, perennial lineages exhibit a unique and highly diversified repertoire of *NLR* genes with limited interspecies synteny. The investigation of gene gain and loss ratios revealed that this diversification resulted from the birth of novel genes following individual speciation events. Among perennials, *G. latifolia*, a well-known resistance resource, has the highest ratio of these novel genes in the tertiary gene pool. Our study suggests evolutionary mechanisms, including recombination and transposition, as potential drivers for the emergence of these novel genes. This study also provides evidence for the unbalanced expansion of the *NLR*ome in the D^t^ subgenome compared with the A^t^ subgenome in the young allopolyploid *G. dolichocarpa*. To the best of our knowledge, this is the first study to investigate the effect of annuality and perenniality life transition on the evolution of *NLR* genes in the genus *Glycine* to identify its genomics resources for improving the resistance of soybean crop with global importance on the economy and food security.

## Introduction

Soya (*Glycine max*), a legume, is the fourth most important agricultural commodity in the world. It is widely cultivated and used to produce biodiesel, high-quality livestock feed and the cheapest edible protein for humans (Sedivy et al., 2017). The genus *Glycine* comprises two subgenera (*Glycine* subgenus and *Soja* subgenus) that diverged approximately 5 million years ago (Mya) and exhibit different life strategies. The subgenus *Soja* consists of annual plants such as the soybean (*Glycine max*) and its wild ancestor*, Glycine soja*, which was domesticated about 6,000–9,000 years ago. These plants are indigenous to eastern Asia, including China, Japan, Korea, and some parts of Russia. In contrast, the subgenus *Glycine* encompasses around 30 perennial species. The majority of these species are found in Australia and inhabit a variety of environments, including deserts, sandy beaches, rocky outcrops, as well as monsoonal, temperate, and subtropical forests (Innes et al., 2008). All diploid *Glycine* species have a chromosome count of (2n = 4x = 38 or 40), which is different from most species in the legume tribe *Phaseoleae*, which usually have (2n = 20 or 22). Both *Glycine* subgenera basically have (2n = 40). More recently, probably within the past few tens of thousands of years, allopolyploid taxa have appeared within the subgenus *Glycine*. These freshly developed allopolyploids of the *Glycine* subgenus have been discovered as far north as Taiwan and the Ryukyu Islands (Egan and Doyle, 2010; Forrester and Ashman 2018). The progenitor of the entire *Glycine* genus underwent a polyploidy event approximately 5–13 million years ago. Within the last 500,000 years, an additional wave of genome duplication has occurred in the subgenus *Glycine*, giving rise to a vast and fully characterized polyploid complex (Sherman-Broyles et al., 2014; Kim et al., 2015). This complex has eight allopolyploid species (2n = 78, 80) and one autopolyploid species (2n = 80), which were produced from diverse combinations of diploid (2n = 38, 40) genomes. Among them, *G. dolichocarpa* (4n=80) is a tetraploid that consists of two subgenomes A^t^ and D^t^, which arose from two progenitor species *G. syndetika* (A) and *G. tomentella* (D) (Li et al., 2014; Manzoor et al., 2019).

A reference genome for the cultivated soybean (*G. max*) accession ‘Williams 82’ was released in 2010 (Schmutz et al., 2010), while reference genomes for its annual relative G. *soja* accession IT182932 and W05 were published in 2019 (Xie et al., 2019). The genomes of *Glycine* species were divided into seven categories based on their potential to form fertile hybrids and the degree of pairing of meiotic chromosomes. These groups were labeled with capital letters A through G (Sherman-Broyles et al., 2014; Kim et al ., 2015). The annual wild cousin of soybean (*Glycine soja*) and the cultivated soybean (*Glycine max*) both belong to the G genome group and have the same number of chromosomes (2n = 40).

The perennial wild relatives of soybeans possess significantly more complex genomes compared to their annual counterparts, which are categorized into six distinct genome groups (A–F). Members of the perennials, subgenus *Glycine* have 2n = 38, 40, 78, and 80 chromosomes, in contrast to the subgenus *Soja* (Sherman-Broyles et al., 2014). Perennial soybeans have not had as much genomic study as their annual counterparts, despite efforts to explore their genetic properties.

Among plant resistance genes, nucleotide-binding domain leucine-rich repeat (*NLR*) genes are the principal component of the effector-triggered immunity (ETI) (Cui et al 2015). Through these intracellular *NLR* genes, resistance reactions result in infected plant tissues, typically accompanied by hypersensitivity reactions (HRs) (Pan et al., 2000; Jones et al., 2016). When invading pathogens are detected, these *NLR* proteins become activated, leading to changes in the conformation of the nucleotide-binding site (NBS) domain. To stop infected cells from spreading, the N-terminal domains of the exposed NBS domain start a downstream hypersensitive response (HR) that causes apoptosis to stop the proliferation and transmission of the pathogen (Andersen et al., 2018; Wang and Chai 2020). Based on the type of N-terminal domain, *NLR* genes are divided into four subclasses: Toll/Interleukin-1 receptor-like (TIR-*NLR*), RX-type coiled-coil (CC-*NLR*), CC_R_-*NLR* subclade with RTP8-type CC domain (CC_R_-*NLR*), and G10 subclade (CC_G10_-*NLR*), a recently proposed category with a distinct type of CC that forms a monophyletic group. Given the global dominance of soybeans, there is a growing need to enhance their genetic potential. Both domesticated (*G. max*) and wild (*G. soja*) annual species show lower levels of genetic diversity (Quershi et al ., 2023).

Globally soybean production is significantly hampered due to high susceptibility of *G. max* to viral, bacterial, fungal and nematode pathogens. In the US alone, an estimated loss of $95.48 billion were occurred during 1996 to 2016 highlighting the significant financial implication of these pathogens on soybean production (Bandara et al., 2020). Given the economic importance of soybean and the significant yield losses caused by various diseases, understanding the NLR genes in soybean is vital for crop improvement programs (Araújo et al., 2019). It enables the development of soybean varieties with enhanced disease resistance through traditional breeding or biotechnological approaches. For example, the identification and functional characterization of NLR genes can lead to the development of soybean lines harboring single or multiple NLR-encoding R gene receptors, potentially offering broad-spectrum resistance (Araújo et al., 2019). The perennial wild relatives of the subgenus *Glycine* are a valuable resource due to their disease resistance genes and adaptability to various habitats. To identify the unique mechanism of *NLR* gene evolution, it is necessary to perform a comprehensive characterization of *NLR* genes in both annual and perennial species of *Glycine*. The major objectives of this study to investigate the long-term evolutionary history of NLR genes in annuals and perennials species, identification of novel resistance resources in genus *Glycine* and effect of diploidization on their evolution. This study provides insights into the complexity and evolution of *NLR* genes in *Glycine* species, which could enhance disease resistance in *Glycine* crops and aid in the development of more resilient soybean cultivars.

## Materials and methods

### Mining of *NLR* genes in for *Glycine* species

The genome sequences of various species within the *Glycine* genus can be accessed through several genome database portals. For this study, we prioritized chromosomal anchored genomes and annotation files for all nine *Glycine* species and acquired them from two portals (NCBI and legume-info) database (**Supplementary Table S1**). We downloaded the genomes of *G. max, G. cyrtoloba, G. stenophita, G. dolichocarpa, G. falcata, G. syndetika, G. latifolia, G. tomentella, and G. soja* (**Supplementary Table S1**). To enable comparison of ancestral species, G. *dolichocarpa*, a tetraploid species, was divided into two distinct subgenomes, A^t^ and D^t^. All genome files were labelled into their respective transcriptome, proteome, and gene transfer file formats. The reference proteomes of all nine *Glycine* genomes were processed using the *NLR*tracker pipeline (Jones et al., 2016; Kourelis et al., 2021). The *NLR*tracker produces output files containing sequences of *NLR* and *NLR*-associated sequences, as well as annotations of *NLR*, NBARC, deduplicated NBARC sequences, and domain sequences. These files were generated using interproscan and specified motif patterns. To ensure clarity, each NLR gene was subjected to manual curation using clustering and phylogenetic analysis, given the unclear nature of NLRtracker’s CCR-NLR annotation.

### Phylogeny and classification of *NLR* Genes

A library of NBARC domains has been produced by the PRG database, which includes reference *NLR* genes (Calle García et al., 2022). As previously mentioned, domain clusters were created using UCLUST’s 70% identity criteria (Edgar, 2010). The group category was assigned to each cluster using the subgroup nomenclature by (Seo et al., 2016). Seo et al. (2016) used classified clusters as seed probes to extract the NBARC domains of the *Glycine* genus produced by *NLR*tracker. These domains were then aligned with the NBARC seed probes to facilitate a comprehensive phylogenetic study of *Glycine*. Insights into evolution were obtained using the maximum likelihood technique of IQtree v 2.0 (Nguyen et al., 2015). The best fit model, VT + F + R10, was chosen along with 1000 bootstrap repetitions as the adjusted value.

### Loci maps and syntenic maps of *NLR*-genes

The Interproscan tool was used to generate various output files, such as the GFF3 file, NLR fasta file, and an assembled file for gene density maps. After that, we used an adjusted bin size of 5 kb to intersect NLR gene sequences in the annotated file using BEDtools (Quinlan and Hall, 2010). After completing this procedure, count files were produced, which were subsequently modified by assigning each coordinate a bin number. Using the Rldeogram package, this generated a karyotype file for display in R (Hao et al., 2020).

### Evolutionary analysis in *Glycine NLR*s

As previously stated, Clustalw was used to align the deduced protein sequences of paralogs with their compatible subgroups (Li, 2003). The alignment of the respective nucleotide sequences was performed using the Perl-based pal2nal software (Suyama et al., 2006). For improved alignment, gaps and codons were removed, and Ks values were determined using the MA method and Kaks calculator. Depending on how nucleotide and protein sequences are substituted, Kaks values can be either non-synonymous (changing over time) or synonymous (changing over time).The frequency of evolution in various species may be inferred from Ks values. To prevent substitution saturation, Ks values larger than two were eliminated. *NLR* genes were grouped using the Orthovenn2 program to investigate orthologs. Orthovenn2 discovered common genes across all species by supplying the protein sequences of suspected *NLR* genes of various species with an E-value of 1e-2 as the default option (Xu et al., 2019). *NLR* genes were submitted to Orthofinder for a thorough examination of orthology (Emms and Kelly, 2019). Tree building was performed using the obtained orthologs. The number of gene gain and loss output was obtained sequentially by CAFE5 using orthogroup and ultrametric tree files as input. The available literature provides a thorough overview of evolutionary analysis (Areej et al., 2023).

### Lineage specific gene analysis

Phylogenomic analysis was performed using Notung (Version 2.9) Command Line Interface (CLI). This software was used to calculate the lineage-specific genes (LSGs) in the genus *Glycine*. The gene tree and species tree were reconciled under postfix species labels. Prior to reconciliation, the species tree was converted into a binary tree. Following the phylogenomic analysis, Notung saved the output in a Homolog table. This file contained descriptions of Paralogs, Orthologs, and Xenologs. The data provided by these results were then used to generate an UpSet plot using R. This plot indicated the conserved lineages across different species within the genus *Glycine*.

### Comparative transcriptomics

To perform the expression analysis of identified NLR genes, we utilized available datasets from PRJNA628842 and PRJNA393826 bioprojects using the NCBI database (**Supplementary Table S1**). We aligned the raw read sequences using the reference genome of *G. max* and *G. soja* with HISAT (Pertea et al., 2015, 2016). Alignments were passed to StringTie for transcript assembly. Lastly, Ballgown was used to process the assembled transcripts and abundance to group the experimental conditions and identify the genes that were differently expressed between the conditions (Pertea et al., 2015, 2016).

## Results

### Expanded *NLR*ome in annuals in contrast to perennials species of *Glycine*

**Figure 1:**
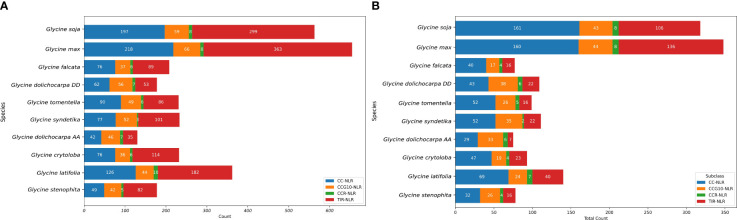
Comparative analysis of *NLR* genes and its subclasses in genus *Glycine:* A nucleotides domain-based perspective **(A)** Distribution of *NLR* genes in major classes across genus *Glycine* including partial and full length *NLR* genes **(B)** Distribution of Intact *NLR* Genes in *Glycine* Species.

Significant differences of *NLR* genes were observed among annual and perennial species. Identified *NLR* genes can be classified into four sub-classes CC-*NLR*, CC_G10_-*NLR*, TIR-*NLR* and CC_R_-*NLR* (**Figure 1A**). *NLR* genes are often incomplete and have undergone pseudogenization due to gene duplication, retro-transposition, non-processed inactivation, and nonsense mutations. We further performed manual filtering for intact *NLR* genes from each class and a similar trend was identified among *Glycine* species (**Supplementary Table S2**) and 318 and 348 NLR genes were found in *G. soja and G. max*, respectively. Relatively contracted *NLR* gene distribution was found in perennials, including *G. falcata* (77), *G. dolichocarpa* (184), *G. syndetika* (111), *G. tomentella*, (99), *G. cyrtoloba* (93), *G. latifolia* (140), and *G. stenophita* (78) (**Figure 1B**). *NLR* genes belonging to sub-class CC-*NLR* and TIR-*NLR* have gained substantial expansions as compared to their distribution among seven perennials’ species. Another subclass, CC_R_-*NLR*, which is a key component in network of *NLR*ome. They remained highly conserved in all members of the family *Fabaceae* and their extent remained conserved, yet their expansion was also observed in annuals. Studying the factors contributing to the increased prevalence of *NLR* genes in annuals compared to perennial species is of immense importance. Recent phylogenomics suggest an increased number of non-redundant genes in annuals (129,006) as compared to perennials (109,827) (Zhuang et al., 2022). Expansion of *NLR* genes in annuals is also consistent with the super-pangenome of *Glycine* that provides evidence of a higher rate of non-core gene formation in annuals as compared to perennials. *G. dolichocarpa* subgenomes (A^t^ and D^t^) have shown contraction of *NLR* genes upon comparison with their progenitor of *G. syndetika* (A) and *G. tomentella* (D). Significant reductions in TIR-*NLR* genes were observed in both A^t^ and D^t^ subgenomes. Interestingly *G. dolichocarpa-D^t^* has shown increased ratio intact *NLR* genes as compared to its progenitor *G. tomentella* (D) in contrast to A^t^ which have shown considerable contraction as compared to *G. syndetika* (A) especially 66% TIR-*NLR* were lost after allopolyploidization. Overall, *NLR*ome in both A^t^ and D^t^ have shown biased subgenomic fractionation as D^t^ have shown higher ratio of intact *NLR* genes as compared to A^t^. Comparing the A, D, A^t^, and D^t^ genomes revealed that the A^t^ and D^t^ subgenomes of the allopolyploid lost 7,351 genes, with a higher number of losses from D (4,109) than from A (3,242) (Zhuang et al., 2022). This suggests that this biased fractionation of *NLR* genes is asymmetrical to the total number of genes lost in each subgenome.

### *NLR* gene density is not a function of genome size

**Figure 2:**
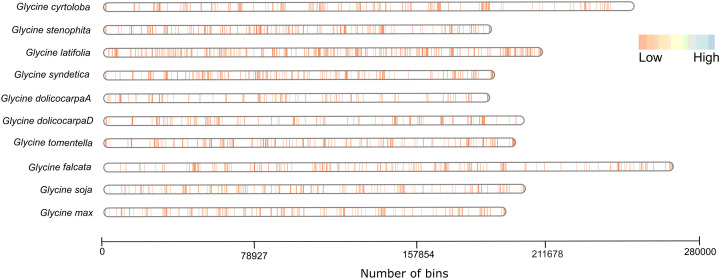
Gene density map: This graph displays bins on the x-axis, each representing a 5kb segment of the genome. The color gradient from brown to blue signifies the varying density of *NLR* genes. This graphical representation provides a quantitative correlation between *NLR* gene density and genomic size of different species of *Glycine*.

We further correlated the *NLR*ome of each species with respect to their genome size and examined the structural organization of *NLR* genes. All genome assemblies anchored at the chromosomal level were analyzed to construct *NLR* gene density maps. A significant diversity of resistant genes was established across the different members of genus *Glycine*. The gene density map (**Figure 2**; **Supplementary Table S3**) demonstrates that the density of *NLR* genes does not correlate with genome size within the genus *Glycine*. For instance, *G. latifolia*, despite having a relatively smaller genome size of 995 MB, exhibits an expanded *NLR*ome. This contrasts with *G. falcata*, which has a larger genome size of 1.39GB and displays a reduced *NLR*ome density. Similarly, *G. cyrtoloba*, with a genome size of 1.3GB, has a constrained *NLR*ome. This is different from *G. syndetika* and *G. stenophita*, which, despite their smaller genome sizes of 948 and 940 MB, respectively, possess a higher *NLR*ome density than *G. cyrtoloba*. Interestingly, there is a contraction of *NLR* gene families in both subgenome A^t^ and D^t^ of the allotetraploid species *G. dolichocarpa* when compared to its ancestors, *G. tomentella* and *G. syndetika*. The contraction of *NLR* gene families in *G. dolichocarpa* suggests that polyploidization events do not necessarily lead to an expansion of gene families but can also result in their contraction. This observation aligns with previous findings that polyploidization can either increase or decrease certain gene families, contributing to the complexity of our understanding of genome evolution (Yin et al., 2019). These findings emphasize the complex dynamics of *NLR* gene density and its independence from genome size, highlighting the complex interaction of genetic factors in shaping the *NLR*ome across different species within the genus *Glycine*. The expanded *NLR*ome in *G. latifolia* and the reduced *NLR*ome in *G. falcata*, despite their contrasting genome sizes, indicate that *NLR* gene density is not merely a function of genome size. Instead, it may be influenced by a multitude of factors, including the species’ evolutionary history, environmental pressures, and genetic mechanisms. We further illustrated that individual gene density maps on each chromosome revealed significant variation in the distribution of *NLR* genes. Annual species like *G. max* and *G. soja* have more pronounced high-density regions (**Supplementary Figure S1**), suggesting a higher concentration of NLR genes, which could be crucial for their immune response. In contrast, perennial species show a more uniform and lower density distribution, indicating fewer NLR genes or a more even spread across their genomes.

### Classification of identified *NLR* genes

**Figure 3:**
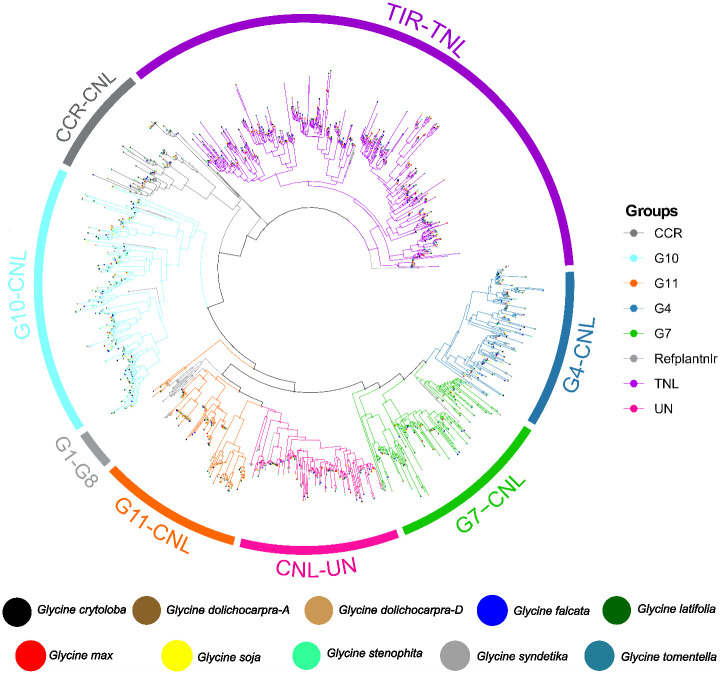
Phylogenetic distribution of 8 genomes and two subgenomes of genus *Glycine* among four major subclasses of *NLR* genes 1) CC-*NLR* 2) CC_G10_-*NLR* 3) CC_R_-*NLR* 4) TIR-*NLR*.

Furthermore, a comprehensive classification of *NLR* genes was performed across the genus *Glycine*. The evolutionary history of *NLR* genes in the genus *Glycine* can be traced by constructing their phylogenetic relationships. This is achieved by analyzing the amino acid sequences of the conserved NB-ARC domain. The TNL clade branched out as expected. The CNL class split into three major subclades: CCR-*NLR*, CC-*NLR*, and CCG10-*NLR*. Within the CC-*NLR* sub-clade, we identified four significant sub-groups: CNL-Un, CNL-G11, CNL-G7, and G4. The TNL clade exhibited polyphyletic traits, suggesting multiple radiations and significant diversification (**Figure 3**). In comparison, CC_G10_-CNL exhibited considerable diversity and expansion with seven radiations. CC_R_-*NLR* doesn’t diversify and remains highly conserved across all species. The complete absence of groups from G1-G8 across the genus *Glycine* aligns with previous findings (Asif et al., 2023, Rani et al., 2023, Areej et al., 2023). The multiple radiations of G7 and G4 also suggest their significant diversification throughout the genus *Glycine*. The expansion and absence of certain groups in genus *Glycine* directly related to the specific pathogen they detect. The encounters of *Glycine* species with different pathogens lead to the diversification of certain groups like we observe in G10, G4 and G7, and the absence of certain pathogens also leads to the lack of associated sub-groups, as in this case G1-G8.

### Contrast in gene birth and lost ratio in annuals and perennials

**Figure 4:**
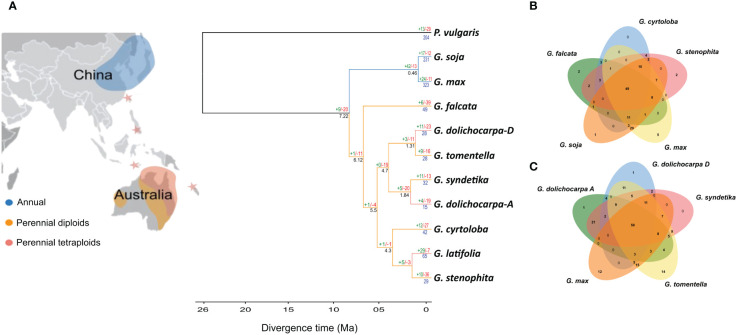
Unraveling gene gains and losses in *Glycine* species: **(A)** The phylogenetic tree visualizes the evolutionary trajectory of *NLR* genes, highlighting gene gain (green), loss (red), and duplication (blue) events across the chronological spectrum. Additionally, the branch color indicates the global distribution of each species, linking genetic evolution to geographical occurrence. **(B, C)** The Venn diagram illustrates the distribution of species-specific and orthologous *NLR* genes across the sub-genus, providing insights into the genetic diversity and evolutionary relationships among the species.

Since we have observed that the annual lineage (*soja*) of *Glycine* have an expanded *NLR*ome as compared to the perennial lineage (*wild*). These expansions in the *NLR*ome are either due to the birth of new genes or duplication of existing genes. The study of gene gain and loss of *NLR* genes in the genus *Glycine* was conducted using Orthofinder and CAFÉ analysis. Family trees for each *Glycine* species and its subgenome were constructed by comparing them with *Phaseolus vulgaris*, its closest allies in the legume tribe. Despite the unique whole genome duplication (WGD) event that occurred in the ancestor of annuals (*soja*) and perennials (wild), a significant loss of *NLR* genes was observed in the ancestral lineage (**Figure 4**). The contraction of *NLR* genes after WGD in *Glycine* is consistent with previous observation that the WGD event is followed by a trend of diploidization leading to contraction. Furthermore, following the divergence from perennials, the annual lineage (*Soja)*, exhibited an expansion of *NLR* gene families (**Figure 4**). The most significant gene gain of 42 gene families were observed in the ancestral lineage of the subgenus *Soja*. The highest rate of terminal duplication is found in both annual species of the subgenus *Soja*. In contrast, the perennial subgenus *G. wild* demonstrates an overall decrease in the number of *NLR* gene families across the subgenus. The greatest gene loss was observed in G. *falcata*, which lost 39 gene families and gained only 6 of them. Terminal duplication also shows a downward trend across the perennials,except for *G. latifolia*, which gained twenty-nine gene families and lost only seven of them. Considering the evidence from gene birth and death analysis, we can hypothesize that the birth of new sub-gene families, lineage specific and terminal duplication could be the major reason for expanded *NLR*ome in annuals species. We also compared the shared orthologs between perennials and *G. max* (**Figures 4B, C**). In total 49 shared orthologs were present between perennials diploid species (*G. falcata, G. cyrtoloba and G. stenophita*: 4B). Increased number of shared ortholog of up-to 59 were present between *G. max* and polyploid perennials (*G. dolichocarpa, G. tomentella* and *G. syndetika*:4C).

### Evolutionary history of *NLR* gene evolution

**Figure 5:**
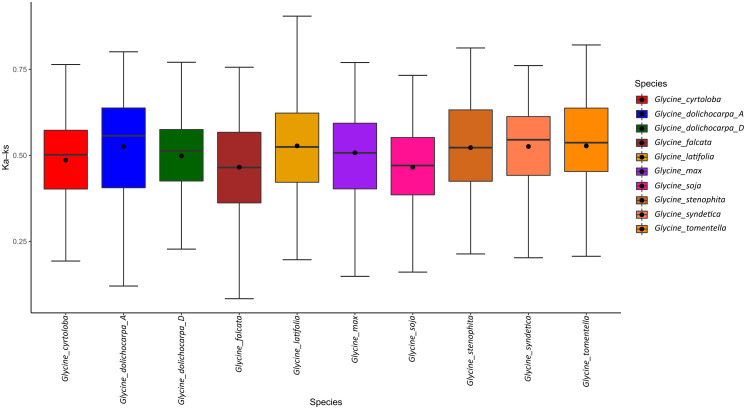
Evolutionary history of *NLR* gene duplication in *Glycine* species.

Considering the immense importance of gene duplication, as illustrated by gene gain and loss analysis. We explored the duplication history of *Glycine* by comparing *Ks* values between paralogs of each subgroup. The closest estimates suggest that divergence between annuals and perennials occurred at ~7 Mya. Collective Ks values obtained from all members of the genus *Glycine* have shown a common duplication curve since 26 Mya after the split from the recent common ancestor *Phaseolus vulgaris*. Substitution analysis further suggests that the rate of duplication increased after the *Glycine*-specific whole-genome duplication (WGD) event, which occurred approximately ~10 Mya (**Figure 5**). Despite contraction after ongoing diploidization, the perennials lineage underwent a high ratio of gene duplication between an estimated time range of 3–8 Mya (*Ks*= 0.05–0.01) followed by gradual reduction at the end of this marked increases in the duplication of CC_G10_-*NLR* and TIR-*NLR* were observed during this accelerated gene duplication cycle. However, annuals continued their gene duplications until recently, and the highest duplication rate was observed between 0.1–0.5 Mya. This suggests that annuals species of *Glycine* remained expanding their *NLR*ome and the highest duplication occurred after the divergence of *G. max* and *G. soja* (annuals speciation ~ 0.47 Mya). Interestingly, both annuals’ species *G. max* and *G. soja* have showed pronounced duplications of G4-CNL subgroup that ultimately led to diversification and expansion of CC-*NLR* genes. In short, recent gene duplication is responsible for the expansion of the *NLR*ome in annuals. Secondly, the duplication assay does not provide evidence of recent and accelerated gene duplication rates in subgenomes *G. dolichocarpa*, which is consistent with lower terminal duplication rates provided by Orthofinder analysis. It further suggests that the D^t^-subgenome of *G. dolichocarpa* could have expanded through processes other than gene duplication, such as recombination and transposition.

### Conservation of lineage and species-specific gene

**Figure 6:**
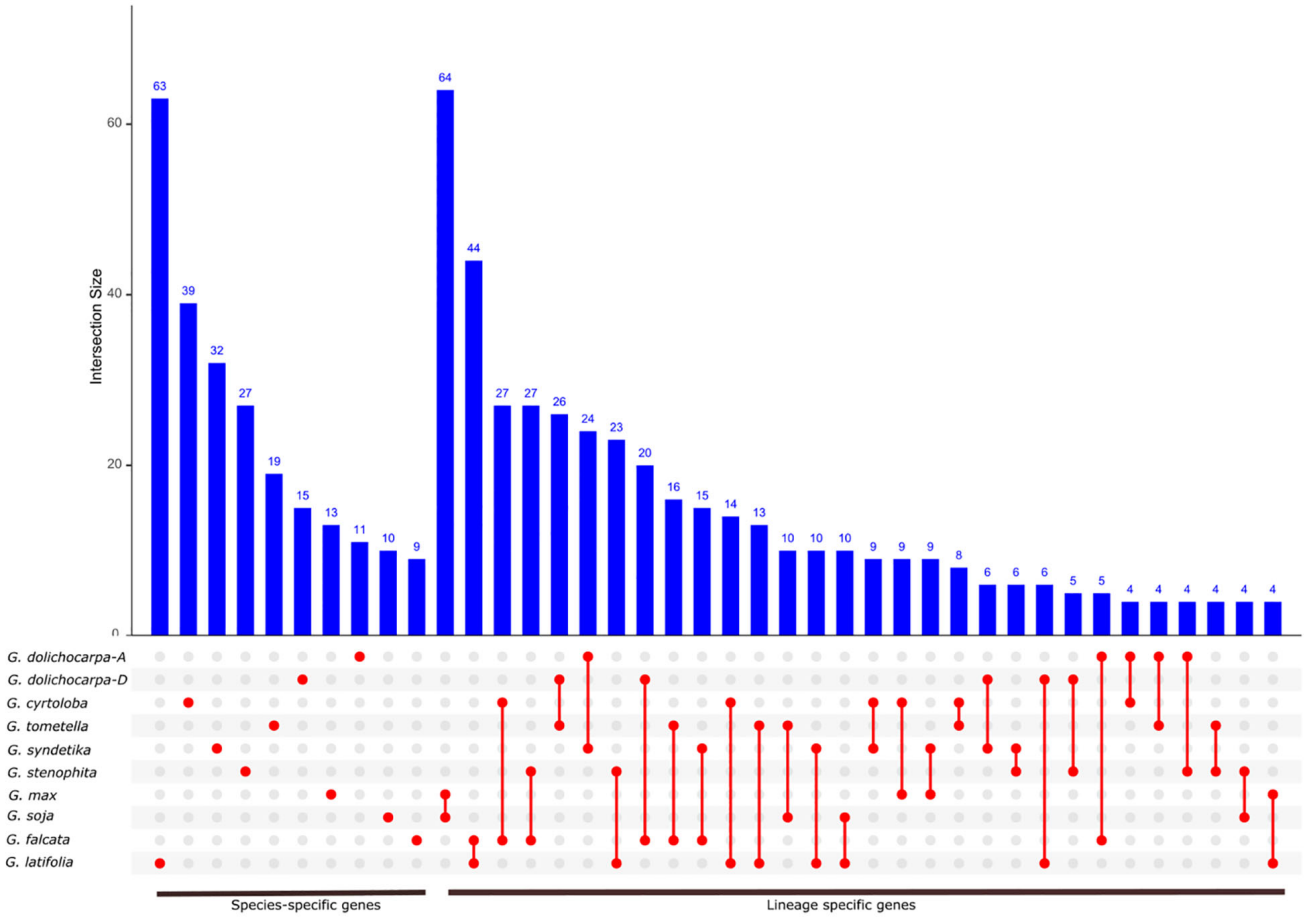
Identification of species and lineage specific *NLR* genes in genus *Glycine*: The blue bars denote the count of lineage-specific genes within each species. The red dots and lines below the bars highlight the lineages that are conserved across multiple species.

We further evaluated the conservation of lineage-specific gene across genus *Glycine* by comparing *NLR* gene tree and species tree using Notung tool. The highest conservation of lineage-specific genes was found among annuals (*G. max* and *G. soja*) where 64 both species shared 64 orthologs (**Figure 6**). Among perennials highest lineage conservation were found among *G. latifolia* and *G. falcata* which is contrast with their relatively earlier divergence (~6 Mya). Furthermore, *G. falcata* also showed a significant share of common orthologs with all members of perennials, suggesting the highest conservation of ancestral *NLR* genes. Comparison of perennials and annuals revealed ten or less than ten conserved lineages. This significant lack of conserved lineages can be explained by the geographical isolation of perennials species since most perennials are native to Australia and annuals are native to eastern China. In the case of tetraploid *G. dolichocarpa*, both subgenomes A^t^ and D^t^ have shared 24 and 26 *NLR* genes lineages with their progenitors *G. syndetika* (A) and *G. tomentella* (D). The highest number of species-specific lineages was observed in *G. latifolia*, which is consistent with the rapid birth of genes and terminal duplication discussed earlier (**Figure 4**). Overall, a higher ratio of species-specific genes was found in perennials and annuals, demonstrating less complex specific *NLR* genes repertoire.

### Synteny analysis

**Figure 7:**
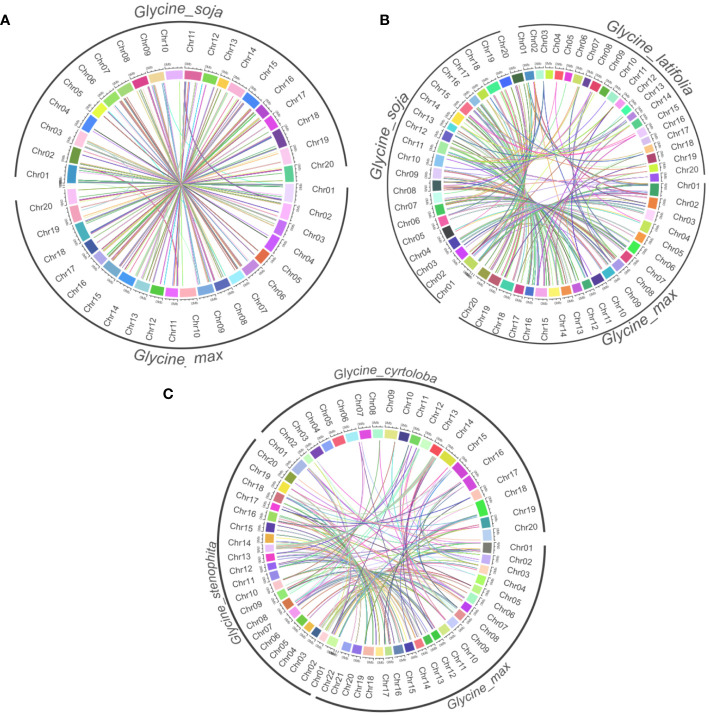
Synteny analysis of *NLR* genes among annuals and perennials species. Each panel represents syntenic relations among **(A)** annuals (*G. max* and *G*. *soja*) **(B)** Annuals species and *G*. *latifolia* **(C)** *G*. *dolichocarpa*, *G*. *max* and *G*. *latifolia.*.

Considering the highest degree of lineage-specific genes among annuals and lower conservation among perennials, we further performed an in-depth comparison by using synteny analysis. It provides a deep understanding of the evolutionary connections, genomic modifications, and preserved functions across various organisms. Among annuals, a considerable amount of syntenic links were discovered among these species (**Figure 3**). This suggests a high level of genomic conservation between *G. max* and G. *soja*, which is expected given their close evolutionary relationship within the annuals. *G. soja* and *G. max* have shown the greatest number of ortholog clusters throughout the chromosomes (**Figure 7A**). We further compared annuals (*G. max* and *G. soja)* and perennial member *G. latifolia. G. max* and *G. soja* exhibit a significant degree of synteny between them, which contrasts with *G. latifolia* (**Figure 7B**). The latter demonstrates a declining trend in the syntenic relationship between orthologs within the subgenus *Soja*. Further, *G. soja* was substituted with the perennial *G. dolichocarpa*. Interestingly, the members of the subgenus G. *wild* did not exhibit as much synteny among themselves as we found within the subgenus *Soja* (**Figure 7C**). Major clusters were identified on chromosomes 1, 2, 3, 18 and 20. High syntenic relation between *G. max* and G. *soja* indicate that they both share a common ancestor, and a large part of the genome has been maintained over time, which shows high genomic stability.

### Comparative transcriptomics of identified *NLR* genes

**Figure 8:**
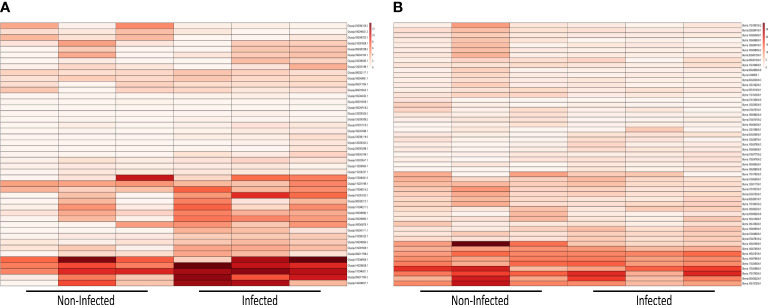
Expression analysis upon *Fusarisum oxysporum* infection **(A)** heatmap of *NLR* genes expressions in infected and non-infected *G*. *soja* **(B)** heatmap of *NLR* genes expressions in infected and non-infected *G*. *max*.

We further evaluated the expression identified *NLR* genes using available datasets. We performed a comparative transcriptomic analysis of both *G. soja* and *G. max* in response to infection by *Fusarium oxysporum* (**Figure 8**). Within the 45-day duration, a total of 45 genes were expressed in *G. soja*, whereas 53 genes were expressed in *G. max*. These genes were categorized into distinct groups: CCR, G10, G11, TNL, and CNL-UN. Notably, the expression of *NLR* genes was significantly higher in *G. soja* (wild soybean) compared to *G. max* (cultivated soybean). Specifically, under both infected and non-infected conditions in the wild, genes such as Glysoja.17G046920.1, Glysoja.14G038028.1, Glysoja.17G046921.1, Glysoja.05G011704.3, and Glysoja.14G038027.1 exhibited elevated expression levels in infected conditions, whereas their expression was comparatively lower in non-infected conditions. These highly expressed genes primarily belonged to the CC_R_-*NLR* and TIR-*NLR* groups. Contrastingly, *G. max* has shown opposite expression pattern. *NLR* gene expression was higher under non-infected conditions compared to infected conditions. Genes such as Glyma.16G215100.1, Glyma.14G079600.1, Glyma.17G245500.1, Glyma.17G245600.1, Glyma.17G179200.1, Glyma.05G082200.1, and Glyma.16G137200.1, categorized under CC_R_-*NLR* and TIR-*NLR* groups, demonstrated this differential expression pattern. Additionally, Glyma.14G079500.1 and Glyma.16G210600.1 exhibited notably high expression levels, specifically in non-infected conditions. Overall, the analysis indicates that the expression of *NLR* genes is substantially higher in wild soybean species compared to cultivated soybeans in response to *Fusarium oxysporum*. Furthermore, distinct expression patterns were observed between the two species under both infected and non-infected conditions, with different sets of genes displaying varied expression levels in response to the pathogen.

**Figure 9:**
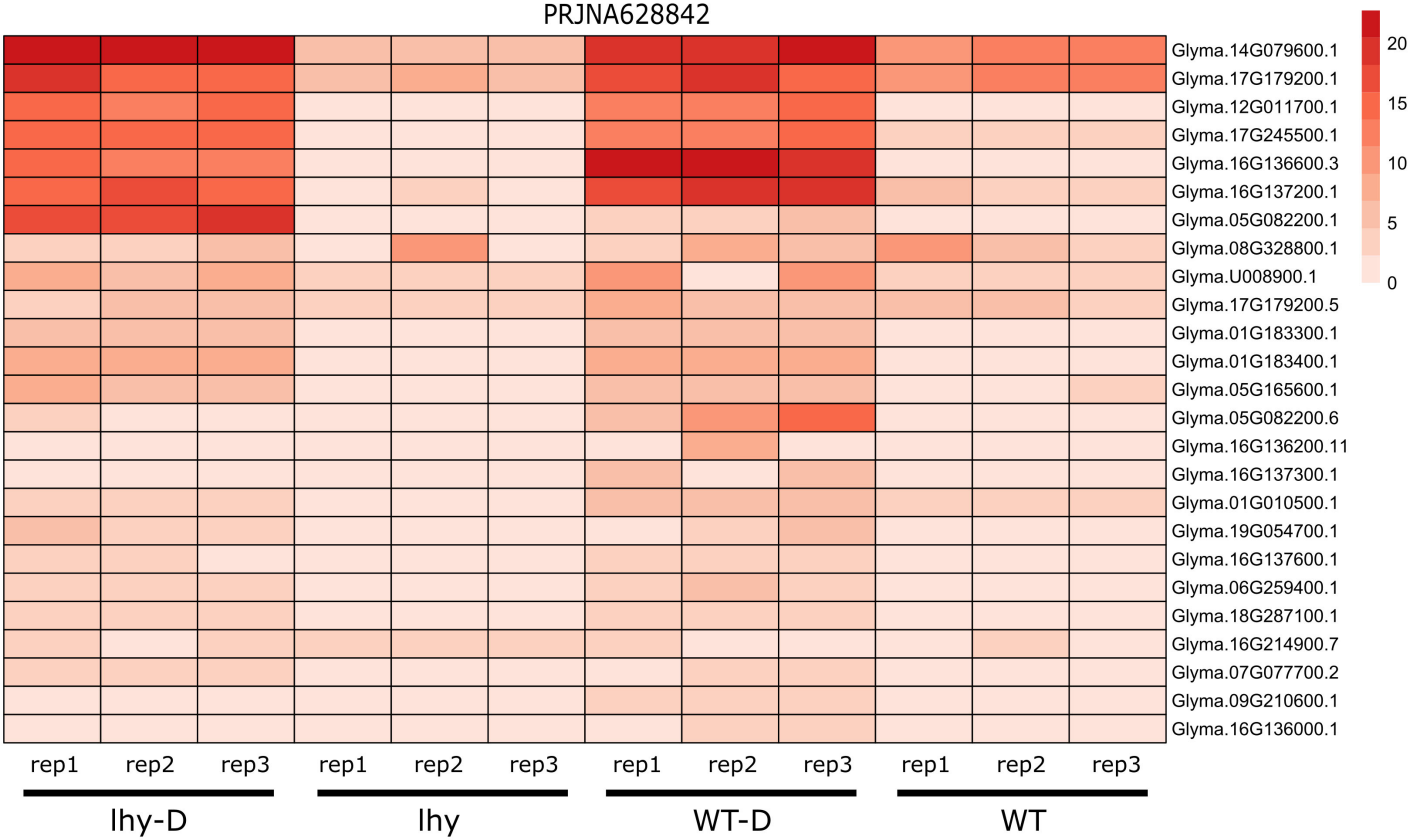
Expression analysis of *NLR* genes upon drought treatment in wild and transgenic *G. max*. LHY lines overexpression LHY genes illustrates higher expression of *NLR* genes drought conditions.

In the PRJNA628842 dataset, the seeds of two lines of *G. max* (LHY and WT) were grown in the Hogland Solution for two weeks and these samples were collected according to the ZT (zeitgeber time) (**Figure 9**). LHY is a transgenic line, that overexpresses Late Elongated Hypocotyl gene (LHY) which is responsible for the circadian movement of leaf in plants (Wang et al., 2021). We performed comparative transcriptomics between two lines of *G. max* LHY and WT and their response to drought stress (LHY-D and WT-D). In normal conditions relatively lower expression of 25 *NLR* genes were observed that can be classified into 5 groups (CCR, G10, G11, TNL and UN), highest number of genes that were expressed belonged to helper *NLR* (CC_R_-*NLR*). However, under drought conditions in LHY and WT, the expression rate of *NLR* genes were higher as compared to well-watered conditions. In total 7 genes have higher rate of expression in both LHY-D and WT-D condition belonging to CC_R_-*NLR*, TIR, and G11-CNL subgroups.

## Discussion

Soybean, originally from China, is now the world’s most widely grown oil and protein seed crop. Despite its agricultural importance, the primary gene pool of soybean, which includes *G. max* (cultivated soybean) and *G. soja* (wild soybean), exhibits low genetic diversity. This limited diversity is a significant constraint on the crop’s environmental resilience and yield potential. *G. max* is particularly susceptible to major pathogens, including viruses, bacteria, and fungi (Araújo et al., 2019; Bandara et al., 2020). Nucleotide-binding site leucine-rich repeat (NLR) genes play a crucial role in plant immunity by recognizing pathogen effectors and triggering defense responses.

### Expansion of NLRome in annuals

Previous study for the characterization of NLR genes was limited to annual species (*G. max* and *G. soja*) and due to fragmented genome assemblies limited number of NLR gene were identified. For example, reduced NLR were identified from *G. max* (Zheng et al., 2016; Afzal et al., 2022). Utilization of updated reference genome and mining approaches has allowed detailed understanding of NLR gene evolution. Our study reveals that both *G. max and G. soja* have an expanded repertoire of NLR genes compared to their perennial relatives. This expansion is attributed to recent gene duplications occurring between 0.1 to 0.5 million years ago in their common ancestor and after their speciation. This is evidenced by a higher ratio of duplicate NLR genes to singletons and the rapid emergence of non-core genes in annuals in annual species compared to perennials (Liu et al., 2018; Zhuang et al., 2022).

### Contraction in Perennials

Perennial species have experienced significant contraction of NLR genes following a whole-genome duplication event approximately 10 million years ago. This contraction has led to a more diversified but smaller set of NLR genes, likely due to gene duplications that occurred between 4 to 7 million years ago after diverging from the annual lineage. It is consistent with previous studies that identified multiple soybean cyst nematode (SCN) population in *G. tomentella* and other species *G. argyria* and *G. pescadrensis* also showed resistance to all tested SCN populations (Wen et al., 2017). Similarly perennial *Glycine* species (*G. argyrea, G. clandestina, G. dolichocarpa, G. tomentella and G. canescens)* have demonstrated resistance to soybean rust (*Phakopsora pachyrhizi*) (Herman et al., 2020).

### Genomic stability in annuals

The high syntenic relationship between *G. max and G. soja* indicates that a large part of their genome has been maintained over time, demonstrating high genomic stability in the annual lineage. Synteny analysis revealed a high degree of genomic conservation between the annual species *G. max and G. soja*, indicating their close evolutionary relationship and shared ancestry. This is consistent with recently constructed *De-novo* assembled pan genome of soybean wild relatives that provide evidence of greater genomic stability of *G. max* and *G. soja* as compared to perennial species and confirmed that large part of genome has been maintained over time (Joshi et al., 2013). In contrast, the perennial species *G. latifolia* exhibited a declining trend in syntenic relationship with the annuals, suggesting divergence within the subgenus Soja. Previous study has also decreased trend of syntenic relation as only 12 out of 12 G. latifolia linkage groups were identified that were colinear with *G. max* chromosomes (Chang et al., 2014). Within the perennial subgenus *Glycine* wild, the species did not exhibit as much synteny among themselves as observed within the annual subgenus Soja. Major syntenic clusters were identified on chromosomes 1, 2, 3, 18, and 20.

### Unique NLR Repertoire in *G latifolia*

*G. latifolia*, known for its high levels of resistance to multiple soybean pathogens and pests, encodes a unique repertoire of NLR genes that are highly species-specific. This diversification might have occurred after the common duplication curve between 4–7 Mya. Despite the ongoing diploidization trend in perennial species, *G. latifolia* has gained 29 gene families with an accelerated terminal duplication rate relative to the closest species (*G. cyrtoloba* and *G. stenophita*). Gene gain and loss analysis provide strong evidence that the birth of new gene families and terminal duplication are significant reasons for the highly divergent evolution of NLR genes in *G. latifolia*. Previous studies highlighted the presence of 3,148 unique sets of genes and noted the overrepresentation of NB-ARC encoding genes (Zhuang et al., 2022). Similarly, comparative analysis with five legume species showed that genes related to defense responses were significantly overrepresented in *Glycine*-specific orthologous gene families (Liu et al., 2018).

### Gene birth and losses is a significant driver of divergent evolution

The evolutionary history of NLR genes in soybean annuals and perennials plants indicates that gene duplications and losses have played a significant role in shaping their current NLR gene profiles. The study of gene gain and loss of NLR genes in the genus *Glycine* revealed that annual species have a higher rate of gene birth and terminal duplication compared to perennials. The most significant gene gain was observed in the ancestral lineage of the subgenus *Soja*, while the greatest gene loss was observed in *G. falcata. G. latifolia*, however, showed a significant gain of gene families, indicating a dynamic evolutionary process. Recently constructed high density linkage maps for *G. latifolia* and their comparison with G*. max* has found significant chromosomal rearrangements in perennial species and annual species *G. max* has undergone more frequent gene duplication contributing to their genetic diversity adaptability (Chang et al., 2014). These dynamics are crucial for understanding how plants adapt to pathogen pressures and environmental changes.

### Conservation of lineage and species-specific genes

The study found the highest conservation of lineage-specific genes among annuals (*G. max and G. soja*), while perennials showed a higher ratio of species-specific genes. This significant lack of conserved lineages between annuals and perennials can be explained by their geographical isolation and different evolutionary pressures.

### Effect of allopolyploidy on NLR evolution

This study provides new insights into the effect of allopolyploidy on the evolution of NLR genes. The availability of the complete genome of A and D diploid progenitors allows a precise definition of its sub-genome and its genome-wide distribution of NLR genes. Comparing the A, D, A^t^, and D^t^ genomes revealed that the A^t^ and D^t^ sub-genomes of the allopolyploid lost 7,351 genes, with a higher number of losses from D (4,109) than from A (3,242) (Zhuang et al., 2022). Conversely, NLR distribution was asymmetrical to the already described complete gene fractionation pattern. Marked expansion of NLR gene distribution was observed for D^t^ as compared to At. This asymmetric expansion of the NLRome in the subgenomes of *G. dolichocarpa* could possibly be due to homologous sequence exchanges (HSEs) and illegitimate recombination. The availability of chromosomal-anchored genome sequences of additional polyploids and their progenitors will further provide a better understanding of the role of polyploids on the evolution of NLR genes.

### Implications for soybean breeding

The findings highlight the importance of leveraging the genetic diversity within the *Glycine* genus to improve soybean’s disease resistance. By identifying and introgressing beneficial NLR genes from wild relatives and other sources, breeders can develop soybean varieties with enhanced resilience and yield potential. This study has significant implications for crop breeding and development of disease-resistance soybean cultivars. The phylogenetic distribution of NLR genes provides a catalog of genetic resources that can be used for crop improvement. By utilizing diverse *Glycine* germplasm, breeders can introduce new resistance genes into elite cultivars, enhancing genetic diversity and crop resilience. The conserved NLR genes are valuable resource for developing broad-specturm disease resistance. G4, G7, G11, CC_R_-CNL and CCG10-CNL perennial lineage-specific NLR genes of *Glycine* offer an opportunity to develop unique disease resistance traits tailored to regional challenges. These genes can be incorporated into breeding programs to create cultivars with enhanced or novel resistance traits. The use of genomic and transcriptomic resources has enabled researchers to uncover the mechanisms underlying the diversification and maintenance of *NLR* gene repertoires, providing valuable insights for future studies on plant immune system evolution and disease resistance. The provided results offer valuable insights into the genetic diversity, evolution, and regulation of *NLR* genes in related plant species, supporting the findings of the study on the genus *Glycine*. These results underscore the broader significance of *NLR* gene research in the context of plant immunity and evolutionary biology.

## Data availability statement

The original contributions presented in the study are included in the article/**Supplementary Material**. Further inquiries can be directed to the corresponding author.

## Author contributions

AS: Data curation, Formal Analysis, Methodology, Software, Writing – original draft, Writing – review & editing. HN: Formal Analysis, Funding acquisition, Project administration, Resources, Visualization, Writing – original draft, Writing – review & editing. FS: Project administration, Resources, Software, Writing – original draft, Writing – review & editing. SN: Data curation, Formal Analysis, Methodology, Writing – original draft, Writing – review & editing. MD: Data curation, Funding acquisition, Methodology, Resources, Software, Writing – original draft, Writing – review & editing. RI: Data curation, Formal Analysis, Methodology, Validation, Writing – original draft, Writing – review & editing. UM: Data curation, Methodology, Software, Visualization, Writing – original draft, Writing – review & editing. AA: Data curation, Investigation, Methodology, Resources, Writing – original draft, Writing – review & editing. NA: Data curation, Funding acquisition, Methodology, Resources, Validation, Visualization, Writing – original draft, Writing – review & editing. RM: Data curation, Methodology, Software, Writing – original draft, Writing – review & editing. SSh: Data curation, Methodology, Writing – original draft. MR: Data curation, Formal Analysis, Funding acquisition, Investigation, Methodology, Software, Visualization, Writing – original draft, Writing – review & editing. SSe: Conceptualization, Data curation, Formal Analysis, Funding acquisition, Investigation, Methodology, Project administration, Resources, Software, Supervision, Validation, Visualization, Writing – original draft, Writing – review & editing.

## Supplementary material

Supplementary Figure 1 Gene density map with respect to each chromosome of *Glycine* species (ranged 1–20). The gradient color is provided to represent the gene density of each chromosome with respect to number bins present in the chromosome.
