# Supplementary figures and images for: Divergent evolution of NLR genes in the genus Glycine: impacts of annuals and perennials’ life history strategies
Source: Front Plant Sci. 2024 Jul 9;15:1383135. doi: 10.3389/fpls.2024.1383135 (PMC11263291; doi:10.3389/fpls.2024.1383135)

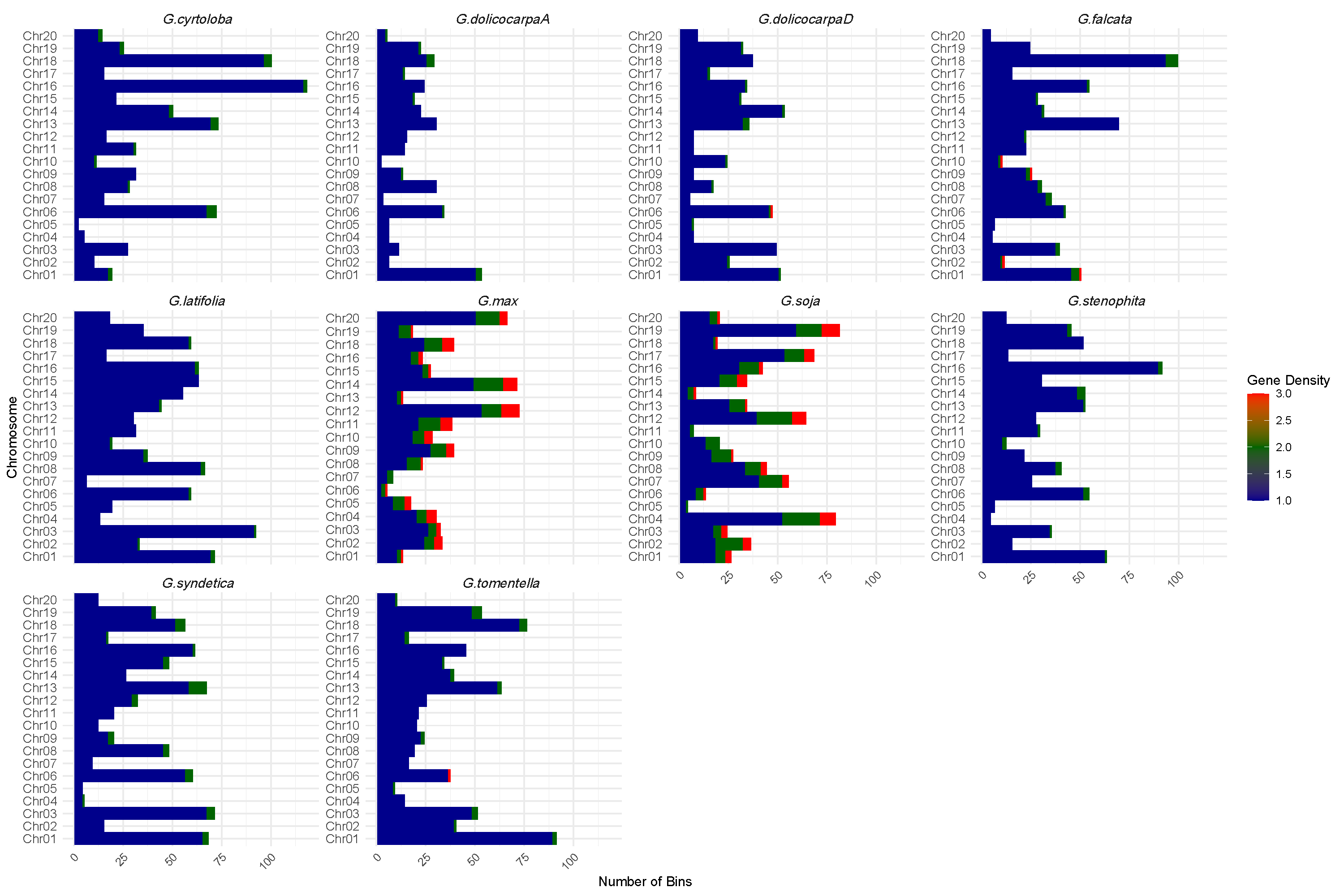

Supplement: Supplementary Figure 1 — Gene density map with respect to each chromosome of Glycine species (ranged 1–20). The gradient color is provided to represent the gene density of each chromosome with respect to number bins present in the chromosome. [file Image_1.jpeg]
